# Comparison of Pharmacokinetic/Pharmacodynamic Parameters and Clinical Outcomes of Vancomycin Administered in Two Different Dosage Regimens in Patients with Acute Kidney Injury

**DOI:** 10.5812/ijpr-159089

**Published:** 2025-04-19

**Authors:** Rokhsareh Soufi, Jamshid Salamzadeh, Ilad Alavi Darazam, Mahdi Amirdosara, Masood Zangi, Minoosh Shabani, Legha Lotfollahi, Zahra Sahraei

**Affiliations:** 1 Department of Clinical Pharmacy, School of Pharmacy, Shahid Beheshti University of Medical Sciences, Tehran, Iran; 2 Department of Infectious Diseases and Tropical Medicine, Loghman Hakim Hospital, Shahid Beheshti University of Medical Sciences, Tehran, Iran; 3 Critical Care Quality Improvement Research Center, Loghman Hakim Hospital, Shahid Beheshti University of Medical Sciences, Tehran, Iran; 4 Department of Nephrology, Loghman Hakim Hospital, Shahid Beheshti University of Medical Sciences, Tehran, Iran

**Keywords:** Vancomycin, Acute Kidney Injury, Pharmacokinetic and Pharmacodynamic (PK/PD) Target, Dose Adjustment

## Abstract

**Background:**

Vancomycin is commonly used in intensive care units (ICUs), but its optimal dosage in patients with acute kidney injury (AKI) remains uncertain. This study aimed to evaluate vancomycin's pharmacokinetic and pharmacodynamic (PK/PD) properties, therapeutic target attainment area under the curve/minimum inhibitory concentration (AUC/MIC ≥ 400) under two dosage regimens, and the need for dosage adjustments in AKI.

**Objectives:**

The primary objective was to assess the proportion of patients achieving a therapeutic AUC/MIC ratio of 400 - 600 mg.h/L and to analyze PK/PD parameters, including volume of distribution (Vd), half-life (T½), and elimination constant (K). The secondary objective was to evaluate kidney function decline, the requirement for renal replacement therapy (RRT), and mortality rates.

**Methods:**

A single-blind randomized clinical trial (IRCT20130917014693N16) was conducted at Loghman Hakim Hospital, Tehran, Iran. Patients over 18 years of age who received at least four doses of vancomycin and developed AKI were included in the study. Eligible patients were randomly allocated into either the intervention or control groups. The intervention group received vancomycin without any dose adjustment, while the control group’s vancomycin dose was adjusted based on daily vancomycin clearance, following the standard protocol in our ICU. Daily serum creatinine, vancomycin peak and trough levels, and clinical outcomes were monitored.

**Results:**

Twenty patients were included in the study. There were no significant differences in baseline data between the two groups. Among the 20 patients, the mean AUC/MIC was higher in the intervention group (345.80 ± 31.95) compared to the control group (251.43 ± 83.10, P = 0.005), although the therapeutic target was not achieved in either group. Despite the lower AUC in the control group, mortality rates, AKI progression, and the need for hemodialysis were comparable between groups (P = 0.29).

**Conclusions:**

Full-dose vancomycin increases the likelihood of achieving PD targets like AUC/MIC in AKI patients. Larger studies are warranted to confirm these findings.

## 1. Background

More than 50% of critically ill and septic patients may experience acute kidney injury (AKI), which can affect patient outcomes in various ways (1-3). Any alteration in kidney function can impact the pharmacokinetic and pharmacodynamic (PK/PD) of many drugs, with antibiotics being among the most important (4, 5). However, there is a significant lack of knowledge regarding antimicrobial dosing in patients with AKI, especially those who do not undergo dialysis. In dialysis, drug clearance is influenced by factors such as the dialysis membrane, flow rate, and duration of dialysis, which differ from AKI without dialysis. In contrast, there is considerable uncertainty surrounding the management of drug dosing in AKI, with most references and guidelines emphasizing the need for frequent drug level evaluations, which can be challenging in a considerable number of countries. Furthermore, current data on dose adjustment is largely derived from studies on chronic kidney disease (CKD) patients, which may not adequately reflect the nuances of AKI.

According to the 2010 kidney disease: Improving global outcomes (KDIGO) conference, a high loading dose (25 - 50% greater than usual) and a normal or near-normal maintenance dosage regimen are recommended for hydrophilic antibiotics (4). Studies on antimicrobial PK/PD in critically ill patients or AKI patients emphasize that even standard dosing recommendations may not be applicable due to fundamental physiologic alterations. The expanded volume of distribution (Vd), especially for hydrophilic drugs, along with reduced protein binding, can significantly alter the drug’s clearance. This suggests that in AKI patients who are not on renal replacement therapy (RRT), careful monitoring and possibly higher dosing may be required to achieve the desired PK/PD targets, such as the area under the curve/minimum inhibitory concentration (AUC/MIC) ratio (3, 6).

Vancomycin, a glycopeptide antibiotic, is frequently prescribed to treat methicillin-resistant *Staphylococcus aureus* (MRSA) infections (7). Previously, therapeutic drug monitoring (TDM) of vancomycin was based on targeting trough concentrations of 15 - 20 mg/L. However, due to a better understanding of the PD profile of vancomycin (from concentration-dependent to concentration- and time-dependent), the 2020 vancomycin consensus guidelines recommend targeting an AUC/MIC ratio of 400 - 600 mg.h/L instead of relying on trough concentrations alone (8, 9). In adults with normal renal function, vancomycin has a half-life (T½) of approximately 7 to 9 hours, with intermediate protein binding (about 55%) and a moderate Vd (about 0.7 L/kg) (10). Since vancomycin is primarily eliminated through the kidneys via glomerular filtration, renal function significantly affects its PD profile and therapeutic success (11).

Despite these recommendations and due to the potential for vancomycin PK/PD alterations in patients with AKI, evidence suggests that adjusted dosing in critically ill patients is often insufficient. This increases the risk of subtherapeutic antibiotic levels, antimicrobial resistance, and poor clinical outcomes. The lack of studies on the PK/PD properties of vancomycin in AKI patients who do not require RRT highlights a significant evidence gap in the literature.

## 2. Methods

### 2.1. Study Design

A single-blind randomized clinical trial was designed and conducted at Loghman Hakim Hospital, affiliated with Shahid Beheshti University of Medical Sciences (SBMU), Tehran, Iran, from September 2022 to August 2023. This study was approved by the Research Ethics Committee of the School of Pharmacy, SBMU (IR.SBMU.PHARMACY.REC.1401.140) and registered in the Iranian Registry of Clinical Trials (IRCT20130917014693N16).

Patients were considered eligible for inclusion if they met all of the following criteria: (1) Age over 18 years; (2) receiving at least four doses of vancomycin; (3) experiencing ongoing AKI due to any cause, as defined by KDIGO criteria: An increase in serum creatinine of at least 0.3 mg/dL within 48 hours, or a decrease in urine output to less than 0.5 mL/kg/h for at least 6 hours; (4) not being pregnant or breastfeeding.

Patients were excluded if: (1) Acute kidney injury resolved; (2) they required RRT for any reason; (3) experienced infection progression while on vancomycin and (4) any unplanned changes in vancomycin dosing, or withholding during the study period

### 2.2. Intervention

With an assumption of an alpha of 0.05 and a power of 80%, 14 patients in each group were calculated as follows (Equation 1):


$$N=\frac{{\left[\left(Z_{1-\frac{\alpha }{2}}\right)+\;\left(Z_{1-\beta }\right)\right]}^{2}\;\times 2}{{∆}^{2}}$$

At the time of AKI detection, eligible patients were randomized into two groups (simple randomization): Intervention and control, with 14 patients in each group, and were followed for three to five days. According to hospital protocol, patients with AKI were to receive adjusted vancomycin doses based on clearance, which was determined using the estimated glomerular filtration rate (eGFR) calculated by the Cockcroft-Gault (C-G) equation (equations 1 - 3). Patients in the intervention group received full doses of vancomycin based on clinical indication (ranging from 40 to 60 mg/kg/day), while participants in the control group received adjusted doses based on calculated vancomycin clearance in the study, which is not a routine approach for dosing adjustment and contributes to the novelty of the study (formulas are provided in equations 1 to 3).

Patients were monitored daily for changes in serum creatinine to confirm the presence of AKI and assess the need for dosing adjustments in the control group. Vital signs, vancomycin adverse effects (nephrotoxicity, ototoxicity, thrombocytopenia, or infusion-related reaction), and clinical outcomes, primarily mortality and the need for RRT, were also monitored throughout their ICU stay. After three days of enrollment, a blood sample was drawn to measure vancomycin peak (1 hour after the end of infusion) and trough (30 minutes before the next dose) concentrations. Vancomycin serum levels were measured using the ARCHITECT-i vancomycin kit, based on a one-step in vitro chemiluminescent microparticle immunoassay (CMIA) (Equations 1 - 3).


$$eGFR\;\left(mL/min\right)=\frac{\left(140-age\right)\left(weight\right)}{srcr\times 72}\times (0.85\;if\;female)$$

$$Vancomycin\;clearance\;\left(l/h\right)=\left\{\left(eGFR\times 0.689\right)+\;3.66\right\}\times 0.06$$

Vancomycin dose=determined AUC×vancomycin clearance

### 2.3. Primary Outcome

The primary objective was to assess the proportion of patients achieving a therapeutic AUC/MIC ratio of 400 - 600 mg.h/L and to analyze PK/PD parameters, including Vd, T½, and elimination constant (K). The formulas for calculating PK/PD parameters are provided in Appendix 1 in Supplementary File (12).

### 2.4. Secondary Outcome

The worsening of kidney function, the need for RRT during ICU hospitalization, and mortality rate were defined as the secondary outcomes.

### 2.5. Statistical Analysis

The normality of quantitative variables was evaluated using the Kolmogorov-Smirnov (K-S) test. An independent samples *t*-test was performed to compare mean values between the two groups. Statistical analysis was conducted using SPSS version 26.0, with a P-value < 0.05 considered statistically significant.

## 3. Results

During the 12-month study period, 982 patients were admitted to the ICU, 307 patients developed AKI, and 41 patients were recruited for the study. The CONSORT chart is provided in Appendix 2 in Supplementary File. Twenty-one patients were excluded based on the inclusion and exclusion criteria, and the remaining participants were randomized into two study groups. Vancomycin was primarily prescribed to treat pneumonia in both study groups, and there was no significant difference between the two groups regarding other indications. In this trial, AKI was defined using the KDIGO criteria, which include an increase of at least 0.3 mg/dL within 48 hours or a decrease in urine output to less than 0.5 mL/kg/h.

Statistical analysis also showed no significant differences between the two groups in terms of demographic characteristics, stages of AKI, and baseline renal function parameters, as shown in Table 1. The mean ± SD values of vancomycin PK parameters for both groups are presented in Table 2. The vancomycin trough concentration was significantly higher in the intervention group compared to the control group, with values of 17.09 ± 3.53 mcg/mL and 12.14 ± 4.82 mcg/mL, respectively (P = 0.03). Although the peak concentration was also statistically higher in the intervention group (42.59 ± 2.38 mcg/mL vs 32.41 ± 9.59 mcg/mL, P = 0.005), the rate of worsening kidney function or the need for RRT was not significantly different between the two groups.

**Table 1.** Baseline Demographic Data, Renal Function and Vancomycin Indication ^a^

Parameters	Intervention Group	Control Group	P-Value
**Age (y)**	58.00 ± 18.18	66.22 ± 11.91	0.37
**Sex **			0.28
Female	5 (45.5)	2 (22.2)	
Male	6 (54.5)	7 (77.8)	
**Weight (kg)**	73.00 ± 10.29	76.56 ± 9.45	0.60
**Creatinine clearance (mL/min)**	87.04 ± 18.99	71.21 ± 22.89	0.14
**AKI stage**			0.29
Stage 1	9	9	
Stage 2	2	0	
Stage 3	0	0	
**Vancomycin indication **			0.53
Pneumonia	7 (63.64)	6 (66.67)	
Meningitis	1 (9.09 )	2 (22.22)	
Sepsis	2 (18.18)	0 (0.00)	
Empiric	1 (9.09)	1 (11.11)	

Abbreviation: AKI, acute kidney injury.

^a^ Values are expressed as No. (%) or mean ± SD.

**Table 2.** Pharmacokinetic Parameters ^a^

PK Parameter	Intervention Group	Control Group	P-Value
**Trough concentration (mcg/mL)**	17.09 ± 3.53	12.14 ± 4.82	0.03
**Peak concentration (mcg/mL)**	42.59 ± 2.38	32.41 ± 9.59	0.005
**K**	0.08 ± 0.01	0.09 ± 0.04	0.30
**T½ (h)**	8.82 ± 2.01	8.71 ± 5.21	0.95
**Vd (L)**	81.77 ± 15.74	67.41 ± 33.03	0.25
**Vancomycin clearance (L/h)**	6.61 ± 1.57	6.03 ± 3.24	0.63
**AUC mg.h/L**	345.80 ± 31.95	251.43 ± 83.10	0.005

Abbreviations: PK, pharmacokinetic; K, elimination constant; Vd, volume of distribution; T½, half-life.

^a^ Values are expressed as mean ± SD.

The calculated AUC/MIC was higher in the intervention group than in the control (345.80 ± 31.95 mg.h/L vs 251.43 ± 83.10 mg.h/L, P = 0.005). One patient in the intervention group achieved an AUC/MIC greater than 400 mg.h/L. However, most of the patients in both groups failed to reach the target therapeutic range of 400 to 600 mg.h/L (7 patients in the intervention group vs all patients in the control group). Statistical analysis showed no significant differences between the groups for other PK parameters. Furthermore, as mentioned in Table 3, statistical analysis revealed no significant differences between the groups in terms of mortality rate and the need for RRT.

**Table 3.** Secondary Outcomes ^a^

Secondary Outcome	Intervention Group	Control Group	P-Value
**Hemodialysis**	2 (18.18)	0 (0.00)	0.29
**Creatinine (mg/dL)**			
First day of study	1.74 ± 0.31	1.64 ± 0.53	0.08
Fifth day of study	2.05 ± 1.13	1.84 ± 0.69	0.82
**Death **	4 (36.36)	3 (33.33)	0.63

^a^ Values are expressed as No. (%) or mean ± SD.

## 4. Discussion

This study was a randomized clinical trial that, for the first time, evaluated the PKs of vancomycin following the administration of two different dosing regimens in patients with AKI who did not undergo dialysis. Given the insufficient data on appropriate vancomycin dosing in AKI and existing studies suggesting the need for higher antibiotic concentrations in these patients, we aimed to assess the PKs of vancomycin in AKI patients. Most previous studies have focused on patients receiving vancomycin while undergoing RRT. The PK/PD properties of antibiotics in critically ill patients with AKI differ from those in patients with CKD or healthy individuals. Factors such as endothelial dysfunction, secondary capillary leak, and the administration of large volumes of fluids and blood products can increase the Vd and proportionally prolong the drug’s T½ in AKI patients. To date, no reliable consensus or studies confirm whether dosage adjustments are needed in these patients (13-15).

In our study, patients in the intervention group, who received full doses of vancomycin, achieved higher AUC/MIC, trough, and peak concentrations than those in the control group, who received adjusted doses. This highlights the need for increasing vancomycin doses in these patients. As stated earlier, there were no notable differences in the need for RRT or mortality rates between the two groups. This could occur for various reasons, not solely due to the dosage regimen. According to the 2020 IDSA vancomycin guidelines, AUC is considered the preferred therapeutic target over trough concentration due to its association with better clinical outcomes and reduced nephrotoxicity (7). The recommended AUC/MIC range of 400 to 600 mg.h/L is favored for maximizing efficacy while minimizing nephrotoxicity. In this study, patients in the intervention group had higher AUCs compared with those in the control, although the PD target was not achieved in either group. One possible explanation for this could be the twice-daily administration of vancomycin, as recent evidence highlights the time-dependent nature of vancomycin. Another contributing factor could be the prescription of insufficient vancomycin doses, even in patients with normal renal function. Van Der Heggen et al. showed that most patients receiving vancomycin doses of 20 to 40 mg/kg/day had subtherapeutic concentrations even if initiating doses were prescribed correctly (12). Blot et al. conducted a prospective, multicenter PK point-prevalence study investigating PK s and PK/PD target attainment of vancomycin in critically ill patients (16). According to their results, 45% of patients did not reach the minimum trough concentration threshold (≥ 15 mg/L).

Since patients in the full-dose group achieved higher AUCs but still failed to reach the therapeutic range, it can be inferred that dosing vancomycin every 6 to 8 hours, or using continuous infusion, in critically ill patients with AKI could increase the likelihood of attaining the PD target. The PK and PD properties of antibiotics in critically ill patients, especially those with AKI, differ significantly from those in healthy volunteers or CKD patients. Consequently, antimicrobial dose adjustment strategies in AKI patients remain controversial (17). In critically ill patients with sepsis, factors such as vasoplegia, capillary leak, and the administration of large volumes of fluids and blood products can increase the Vd and proportionally prolong the drug’s T½ (18). Unfortunately, there is limited data on antibiotic dosing in AKI patients who are not receiving RRT.

In a clinical trial conducted by Hassanpour et al. on meropenem PK/PD parameters in AKI patients not receiving RRT, patients were divided into two groups: One received full doses of meropenem, and the other received doses adjusted based on the C-G equation. Similar to our study, they concluded neither group achieved the PD target of ≥ 80% fT > 4MIC, suggesting that higher doses may be necessary (19). In 2022, Hughes et al. conducted an observational study on antimicrobial dosing in AKI patients, focusing on gram-negative bacteremia treated with beta-lactam antibiotics (13). Similar to our findings regarding the need for higher doses of antibiotics in critically ill patients with AKI, their results indicated that within the first 48 hours of infection-induced AKI, dose adjustments of beta-lactams may not be necessary. The time to AKI recovery was similar across all patients (16).

For information on antibiotic dosing in critically ill patients with AKI, a review by Lewis and Mueller in 2016 discusses the complexity of dosing due to factors such as increased Vd, hypoalbuminemia, and altered protein binding. In critically ill patients, these changes often necessitate higher doses of antibiotics, including vancomycin, to maintain therapeutic concentrations (6), as supported by our study. Our findings suggest that to achieve the vancomycin PK/PD therapeutic target in AKI patients, a full dose administered preferably every 6 to 8 hours, or via continuous infusion, is needed.

### 4.1. Conclusions

Our findings indicate that the administration of full doses of vancomycin in AKI patients was associated with a higher probability of achieving the PKPD target. Although not all patients in the vancomycin full-dose group achieved the PKPD target, the mean AUC/MIC in this group was significantly higher than in the control group. According to the time- and concentration-dependent PKs of vancomycin, administering full doses of the drug as an infusion or every 6 to 8 hours is required for appropriate PKPD target attainment.

### 4.2. Limitations

The small sample size, primarily due to the narrow and strict inclusion and exclusion criteria, should be considered when interpreting our results. Additionally, our intensive care unit (ICU) at Loghman Hakim Hospital is not a closed system, which allows multiple clinicians to visit patients and make changes to their care orders. This situation complicated patient recruitment and increased the number of dropouts. Our study may be considered a pilot study comparing two different dosage regimens of vancomycin in patients with AKI. There was physician hesitation in administering the recommended full doses of potentially nephrotoxic drugs like vancomycin via a three-times-per-day infusion to AKI patients.

ijpr-24-1-159089-s001.pdf

## Data Availability

The dataset presented in the study is available on request from the corresponding author during submission or after publication. The data are not publicly available due to patients’ confidentiality.

## Clinical Trial Registration Code:

IRCT20130917014693N16.
