# Supplementary material for: Comparison of Pharmacokinetic/Pharmacodynamic Parameters and Clinical Outcomes of Vancomycin Administered in Two Different Dosage Regimens in Patients with Acute Kidney Injury
Source: Iran J Pharm Res. 2025 Apr 19;24(1):e159089. doi: 10.5812/ijpr-159089 (PMC12297031; doi:10.5812/ijpr-159089)

Appendix 1: PKPD formula

$$eGFR\ (ml/min) = \frac{(140 - age)(weight)}{srcr \times 72} \times (0.85\ if\ female)$$

$$Vancomycin\ clearance\ (l/h) = \{(eGFR \times 0.689) + 3.66\} \times 0.06$$

$$Vancomycin\ dose = determined\ AUC \times vancomycin\ clearance$$

Other PD parameters were calculated as below:

$$K = \ln \frac{\left(\frac{Cpeak}{Ctrogh}\right)}{\Delta T_{interval}}$$

$$T_{\frac{1}{2}} = \frac{0.693}{K}$$

$$corrected\ Cp = \frac{Cpeak}{e^{-k(T_{peak} - T_{infusion})}}$$

$$corrected\ Ct = Ctrogh \times e^{-k(T_{interval} - T_{trough})}$$

$$V_d = \frac{\left(Dose \times \left(1 - e^{-KT_{infusion}}\right)\right)}{T_{infusion} \times K\left[corrected\ Cp - \left(corrected\ Ct \times e^{-KT_{infusion}}\right)\right]}$$

Based on available data, if a patient has a low drug clearance and the drug's half-life drug is approximately 6 times the duration of infusion, a limited amount of drug will be eliminated during infusion. Therefore, it may be more practical to use the simpler bolus equation. So, the below formula can be used for AUC24hr calculation (9):

$$AUC_{infusion} = \frac{(corrected\ Cp + corrected\ Ct)}{2} \times T_{infusion}$$

$$AUC_{elimination} = \frac{(corrected\ Cp - corrected\ Ct)}{K}$$

$$AUC_{24hr} = \frac{total\ daily\ doses\ of\ vancomycin}{vancomycin\ clearance}$$

Appendix 2: Consort chart

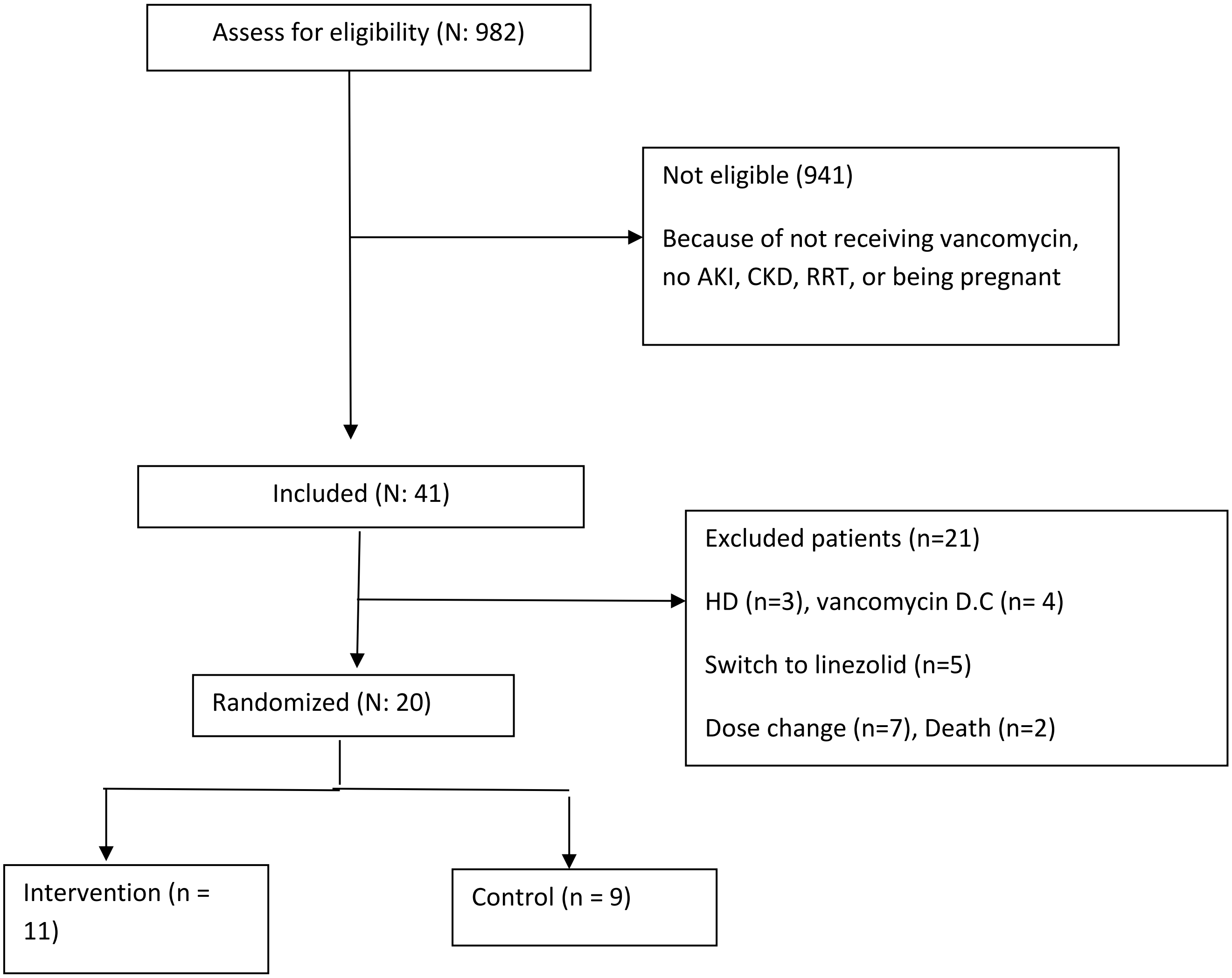

Supplement: ijpr-24-1-159089-s001.pdf [file ijpr-24-1-159089-s001.pdf]
